# Shock, response, and resilience of COVID-19 on Kangaroo Mother Care service utilisation in public health facilities in Bangladesh: An interrupted time series analysis

**DOI:** 10.7189/jogh.14.05014

**Published:** 2024-05-24

**Authors:** Abu Bakkar Siddique, Abu Sayeed, Anindita Saha, Aniqa Tasnim Hossain, Ema Akter, Md Hafizur Rahman, Ashiquzzaman, Syed Moshfiqur Rahman, Anisuddin Ahmed, Shams El Arifeen, Ahmed Ehsanur Rahman

**Affiliations:** 1 Maternal and Child Health Division (MCHD), International Centre for Diarrhoeal Disease Research, Bangladesh (icddr,b), Dhaka, Bangladesh; 2 Global Health and Migration Unit, Department of Women’s and Children’s Health, Uppsala University, Uppsala, Sweden

## Abstract

**Background:**

Premature births and low birth-weight (LBW) are significant contributors to neonatal mortality, particularly in Bangladesh. Kangaroo Mother Care (KMC) has emerged as a proven, safe, and cost-effective intervention to save lives. Nevertheless, the coronavirus disease 2019 (COVID-19) pandemic disrupted health care services, including KMC, and its specific impact on KMC utilisation in Bangladesh remains unknown. Thus, our objective was to evaluate the impact of COVID-19 on KMC utilisation in Bangladesh.

**Methods:**

We conducted a secondary analysis of data, collected from 46 health care facilities in Bangladesh, utilising the District Health Information Systems 2 (DHIS2), spanning from January 2019 to August 2023. Our data set encompassed health care facilities with KMC data points available for at least two months between January 2018 and January 2019. We segmented our analysis to encompass distinct time periods, including pre-COVID-19, lockdown phases, and post-COVID-19. We employed descriptive statistics of KMC utilisation. We performed a segmented regression with a Poisson distribution which was adjusted for autocorrelation and seasonality.

**Results:**

Our study revealed a significant 20% reduction in KMC utilisation during the initial COVID-19 lockdown (incidence rate ratio (IRR) 0.80; 95% confidence interval (CI) = 0.76–0.84) in comparison to the pre-COVID-19 period. However, the 'between lockdown' phase saw a 5% increase in KMC provision (IRR 1.05; 95% CI = 1.01–1.08). Notably, KMC utilisation during the second lockdown resembled pre-pandemic levels, surpassing the initial lockdown period (IRR 0.99; 95% CI = 0.93–1.06). As we transitioned into post-COVID-19 period, KMC utilisation increased by 8% in comparison to the pre-COVID-19 era (IRR 1.08; 95% CI = 1.01–1.16). However, when we considered different facility types, District Hospitals (DH) mirrored the overall trend, whereas Medical College Hospitals (MCH) and Upazila Health Complexes (UHC) facilities exhibited distinctive patterns. Likewise, when assessing divisions, Dhaka, Khulna, and Rajshahi exhibited counter trends to the overall results.

**Conclusions:**

Our study shed light on the profound impact of the COVID-19 pandemic on KMC utilisation, the adaptive responses of health care systems, and the subsequent resilience displayed. District Hospitals played a pivotal role in this rebound. It is essential to recognise that facilities in different divisions were disproportionately affected by the challenges brought forth by the COVID-19 pandemic.

Premature delivery is an immense global concern that results in neonates born with low birth weight (LBW) (birth weight less than 2500 g) [1]. Annually, over 15 million neonates are born preterm (<37 completed weeks of gestation) [2] and an estimated 20.5 million neonates are born with LBW worldwide [3]. These vulnerable neonates account for global neonatal deaths with approximately 17% of all deaths under five-years of age group [4]. In Bangladesh, the preterm birth rate is 19% and the LBW rate is 28%, which accounts for around 11% of all neonatal deaths [1,3,5]. Approximately two-thirds (66%) of preterm neonates die within the first 72 hours of admission [6]. Among those who survive, there are high risks of impaired neurodevelopment and learning disabilities, and other long-term complications [7–10].

A safe, effective, and affordable intervention method known as ‘Kangaroo Mother Care’ (KMC) can prevent these complications and avert preventable deaths [11,12]. Kangaroo Mother Care is defined as early, prolonged, and continuous skin-to-skin contact between the mother or any person and her preterm or LBW newborn, and exclusive breastfeeding or breastmilk feeding [13]. In 2016 a Cochrane review reported on 21 randomised controlled trials (3042 infants) that compared KMC with conventional neonatal care in health facilities and showed that KMC reduced mortality by 40% (relative risk (RR) = 0.60; 95% CI = 0.39–0.92), nosocomial infections by 55% (RR = 0.45; 95% CI = 0.27–0.76) and hypothermia by 64% (RR = 0.34; 95% CI = 0.17–0.67) [14]. It was also reported that KMC increased weight, length, and head circumference, breastfeeding, mother satisfaction with the method of infant care and maternal-infant attachment, and improved child development [14].

The COVID-19 pandemic has brought about significant changes in various aspects of health care. Specifically, it has raised concerns about KMC and breastfeeding practices for mother-newborn pairs [15]. Global recommendations for mother-newborn care during the pandemic are also in conflict, notably with relation to KMC and skin-to-skin contact [15]. However, The American Academy of Pediatrics, the UK Royal College of Obstetricians and Gynecologists, and the World Health Organization (WHO) suggest that women who have COVID-19 should be supported to stay with their newborns and breastfeed, if medically possible, while taking the proper infection control precautions [15–17]. For LMICs, there are not many precise guidelines [15–18].

In 2013, the Government of Bangladesh made a commitment to introduce and expand KMC in health care facilities, with the continuation of care at home [19]. In pursuit of its goal to eliminate preventable child deaths by 2035, the government reaffirmed this commitment in the 'Child Survival Call to Action: A Promise Renewed' [19]. Furthermore, in 2016, KMC was designated as a high-priority intervention for newborn health within the implementation plan of the 4th Health, Population, and Nutrition Sector Programme under the Ministry of Health and Family Welfare [19]. Since the inception of KMC services at health care facilities in 2016, there has been a gradual increase in both the number of facilities offering KMC and the actual provision of KMC services [19]. However, the COVID-19 pandemic disrupted the entire health care system, including KMC practices within these facilities. Given that KMC is primarily delivered within health care settings, the challenges brought about by the COVID-19 pandemic in Bangladesh have likely had a significant impact on KMC utilisation.

To the best of our knowledge, no prior research has examined the specific effects of COVID-19 on KMC practices in Bangladesh. This study was conducted to assess the impact of COVID-19 on KMC utilisation in Bangladesh.

## METHODS

District Health Information Systems 2 (DHIS2) are recommended by the WHO for managing health information in places with few resources. Since 1994, DHIS2 has evolved and is currently used to manage health information in 67 countries [20–23]. DHIS2 has been implemented as a routine data source for collecting, validating, analysing, and presenting aggregate statistical data relevant to integrated health information management activities [24]. This routine data source captures several health-related indicators. District Health Information Systems 2 is a prominent Health Management Information Systems (HMIS) platform in Low- and Middle-Income Countries (LMICs). In 2009, Bangladesh's HMIS implemented the DHIS2 to capture real-time information regarding the use of health services [25]. This covers a majority of public facilities and a limited number of private facilities. We utilised the data extracted from DHIS2 for conducting secondary data analysis. Data was collected on a monthly basis from various public health care facilities in Bangladesh's eight divisions: Dhaka, Chattogram, Barishal, Khulna, Rajshahi, Rangpur, Mymensingh, and Sylhet. These facilities include the Medical College Hospital (MCH), District Hospital (DH), and Upazila (sub-district) Health Complex (UHC). For this analysis, we extracted data from all the health facilities that have provisioned to report KMC in DHIS2 from January 2018 to August 2023. However, we examined the monthly number of babies receiving KMC as reported by the respective health care facilities. We only included facilities that had KMC data points available for at least two months between January 2018 and January 2019. This selection criterion was applied to ensure a cohort of facilities with sufficient and consistent KMC services were included in our analysis. With these inclusion criteria, we included 46 health facilities with 56 months of data from January 2019 to August 2023 in this study.

**Figure 1:**
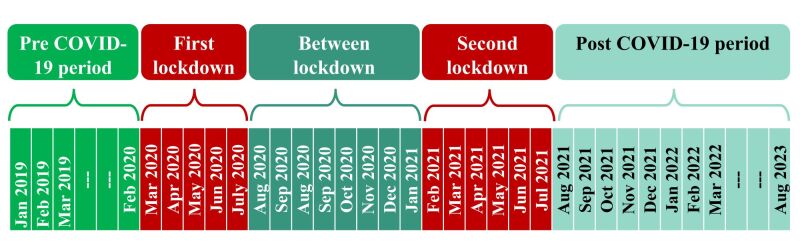
Temporal slices according to coronavirus disease 2019 (COVID-19) response.

We reported findings for various time segments including pre-COVID-19 period, first lockdown, between lockdown, second lockdown and after COVID-19 period (**Figure 1**).

We reported descriptive statistics, which include mean, standard deviation (SD), median, and interquartile range (IQR). We conducted a monthly trend analysis to visually represent the variations in the number of babies receiving KMC from the health facilities, followed by the corresponding predicted values for those months. We applied segmented regression, a statistical method that is frequently employed in interrupted time series (ITS) study. Interrupted time series can be used when we have data about an outcome over time and we want to understand how and if the outcome has changed after an intervention, a policy, or a programme at one specific point in time. By employing the segmented regression, we aimed to capture any changes in the level of KMC occurrences associated with specific periods, such as lockdowns. Segmented regression has been used in many studies to estimate the effect of an intervention on an outcome of interest [26–29]. In addition to that in our data set, the number of KMC variable that is count data, follows Poisson distribution. The application of segmented regression with a Poisson distribution can be performed for analysing count data. The segmented regression model was adjusted to account for autocorrelation and stationarity in order to ensure the accuracy and reliability of our findings. In order to adjust autocorrelation when estimating standard errors, heteroscedasticity and autocorrelation consistent (HAC) standard errors were considered [30]. The seasonality component has been fitted with Fourier series, which are linear combinations of the sine and cosine functions [31]. We reported IRR with corresponding 95% CI from segmented regression for each division and facility type. We reported IRR for first lockdown, between lockdown, second lockdown and after COVID-19 period compared to the reference period that was pre-COVID-19 period. We conducted the analysis using the statistical software package STATA 17.0 MP version (Stata Corp (2017) Stata Statistical Software: Release 15. College Station, TX: StataCorp LLC.).

## RESULTS

**Figure 2:**
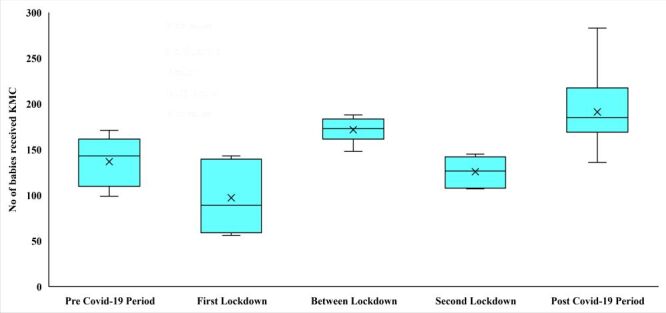
Distribution of number of total Kangaroo Mother Care (KMC) by coronavirus disease 2019 (COVID-19) lockdown.

The distribution of monthly KMC by different phases of COVID-19 based on lockdown periods is presented in **Figure 2**. During pre-COVID-19 period, the median number of KMC per month was 143 (IQR = 51). During the first lockdown period the median number of KMC per month decreased substantially to 89 (IQR = 74). In-between the first and second lockdown, the median number of KMC was higher (173, IQR = 16) than the first lockdown. This number again reduced during second lockdown (126, IQR = 33) and post COVID-19 the number rose up to highest (185, IQR = 48).

**Table 1** describes the mean and standard deviation as well as median and IQR of monthly KMC by facility type and divisions of Bangladesh by different phases of COVID-19 considering lockdowns. The KMC uptake in Medical College Hospitals is significantly lower than District Hospitals and Upazila Health Complexes. District level hospitals had the highest KMC uptake with monthly average 104 with standard deviation 37.5. The district level hospitals had 88 KMCs per month before COVID-19 which reduced to 68 (SD = ±26) during first lockdown. With interim fluctuation between lockdowns the average number of KMC for District Hospitals increased to 124.5 (SD = ±28). The highest number of KMC uptakes was observed in Chattogram (mean = 60, SD = ±24) followed by Sylhet (mean = 33, SD = ±14). Barishal, Mymenshing and Rajshahi had lowest uptakes with monthly average of three. At Chattogram, the average KMC before COVID-19 was 36.6 (SD = ±8.6), which was increased to 78 (SD = ±18.5). However, the number reduced significantly during lockdown periods.

**Table 1:** Descriptive statistics of Kangaroo Mother Care (KMC) utilisation by facility type and divisions of Bangladesh by different phases of coronavirus disease 2019 (COVID-19)

	Pre COVID period		First lockdown		Between lockdowns		Second lockdown		After COVID period		Total	
	Mean (SD)	Median (IQR)	Mean (SD)	Median (IQR)	Mean (SD)	Median (IQR)	Mean (SD)	Median (IQR)	Mean (SD)	Median (IQR)	Mean (SD)	Median (IQR)
Type of facility												
*MCH*	13.5 (4.0)	14.0 (5.0)	7.0 (5.3)	5.0 (2.0)	11.0 (3.3)	11.0 (5.0)	6.7 (3.0)	7.0 (5.0)	10.5 (5.3)	10.0 (6.0)	10.6 (5.0)	10.0 (7.0)
*DH*	88.3 (18.2)	88.5 (26.0)	68.4 (25.9)	69.0 (47.0)	118.7 (9.2)	118.5 (10.0)	84.5 (15.3)	83.5 (31.0)	124.5 (28.0)	117.0 (35.0)	105.5 (30.5)	104.0(37.5)
*UHC*	34.9 (13.0)	37.5 (20.0)	21.8 (11.9)	18 (16.0)	42.0 (4.6)	43.5 (7.0)	34.5 (5.6)	35.0 (7.0)	56.2 (8.2)	58.0 (12.0)	44.0 (15.1)	45.0 (23.0)
Division												
*Barishal*	3.4 (2.4)	3.0 (2.0)	2.0 (1.4)	2.0 (2.0)	1.8 (1.1)	1.0 (2.0)	1.7 (1.2)	1.0 (2.0)	10.7 (7.0)	9.0 (5.0)	6.9 (6.6)	5.0 (7.0)
*Chattogram*	36.6 (8.6)	38.5 (12.0)	37.4 (15.0)	41.0 (23.0)	76.0 (7.0)	77.0 (8.0)	47.2 (5.0)	49.0 (10.0)	77.6 (18.5)	74.0 (26.0)	60.4 (23.7)	57.0 (35.5)
*Dhaka*	19.6 (5.7)	20.0 (6.0)	11.4 (7.3)	10.0 (14.0)	18.3 (3.3)	18.0 (6.0)	10.8 (2.6)	10.0 (5.5)	14.5 (5.5)	14.0 (6.0)	15.5 (6.0)	15.5 (8.0)
*Khulna*	36.9 (5.4)	37.5 (8.0)	24.8 (6.6)	26.0 (12.0)	29.8 (6.4)	28.5 (5.0)	24.0 (8.6)	25.0 (18.0)	26.5 (5.5)	25.0 (9.0)	29.0 (7.6)	30.0 (9.0)
*Mymensingh*	3.5 (1.8)	3.0 (2.0)	2.6 (2.1)	2.0 (2.0)	7.7 (4.7)	6.0 (5.0)	8.3 (1.8)	8.5 (2.0)	12.2 (3.5)	12.0 (5.0)	8.5 (4.9)	8.0 (8.0)
*Rajshahi*	7.8 (9.2)	6.0 (5.0)	0.0 (0.0)	0.0 (0.0)	2.0 (1.4)	2.0 (2.0)	3.3 (1.5)	3.0 (3.0)	4.6 (2.2)	4.0 (3.0)	5.2 (5.3)	4.0 (3.0)
*Rangpur*	4.7 (3.8)	3.0 (5.0)	1.25 (0.5)	1.0 (0.5)	5.2 (3.2)	5.5 (6.0)	6.7 (2.3)	5.5 (4.0)	8.0 (3.6)	8.0 (4.0)	6.2(3.8)	6.0 (6.0)
*Sylhet*	26.6 (10.3)	24.0 (13.0)	19.2 (14.5)	14.0 (23.0)	32.5 (8.2)	34.5 (14.0)	24.5 (9.1)	25.0 (14.0)	38.0 (9.5)	38.0 (12.0)	31.4 (11.7)	32.5 (15.0)

DH – District Hospital, IQR – interquartile range, MCH – Medical College Hospital, SD – standard deviation, UHC – Upazila Health Complex

**Figure 3:**
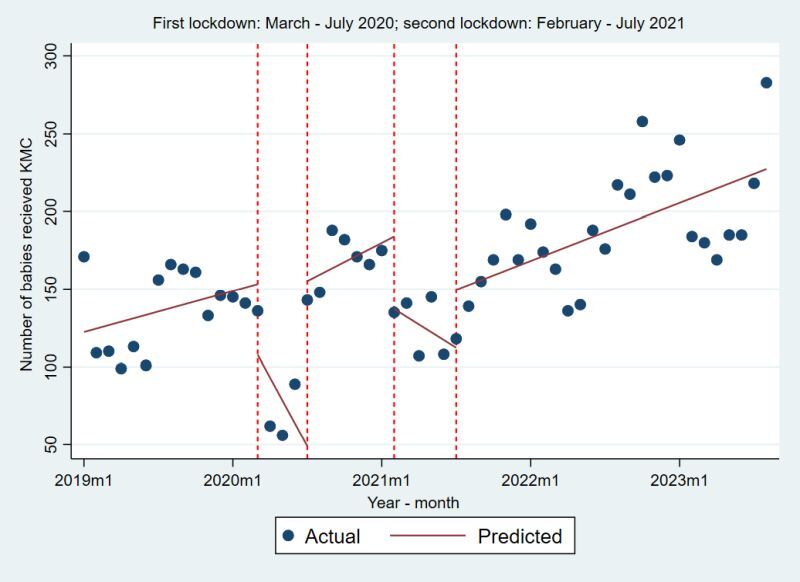
Segmented regression using interrupted time series of number of Kangaroo Mother Care (KMC).

**Figure 3** presents the fitted segmented regression using Poisson link for monthly trend of KMC across different phases of COVID-19 based on lockdown periods. After adjusting for the seasonality and autocorrelation the incidence rate ratio was 0.8 compared to pre-COVID period with 95% CI = 0.76–0.84. After first lockdown and before second lockdown the IRR was 1.05 with 95% CI = 1.01–1.08. There was no significant change in the IRR during second lockdown. After COVID-19 the number of KMC was 1.08 times was higher compared to pre-COVID period with (95% CI = 1.01–1.16). **Table 2** presents the details of the IRR and 95% CI associated with **Figure 3**.

**Table 2:** Results from the segmented regression using interrupted time series of number of Kangaroo Mother Care (KMC)

	Pre COVID period	First lockdown		Between lockdowns		Second lockdown		After COVID period	
	**IRR (95% CI)**	**IRR (95% CI)**	***P*-value**	**IRR (95% CI)**	***P*-value**	**IRR (95% CI)**	***P*-value**	**IRR (95% CI)**	***P*-value**
Bangladesh	Ref	0.80 (0.76–0.84)	0.000	1.05 (1.01–1.08)	0.004	0.99 (0.93–1.06)	0.817	1.08 (1.01–1.16)	0.045
Type of facility									
*MCH*	Ref	0.69 (0.59–0.80)	0.000	0.92 (0.83–1.02)	0.12	0.87 (0.74–1.03)	0.115	0.92 (0.74–1.14)	0.441
*DH*	Ref	0.89 (0.82–0.96)	0.003	1.19 (1.11–1.27)	0.000	1.13 (0.99–1.30)	0.064	1.21 (1.03–1.42)	0.018
*UHC*	Ref	0.61 (0.49–0.77)	0.000	0.77 (0.67–0.87)	0.000	0.69 (0.48–0.99)	0.047	0.74 (0.53–1.04)	0.082
Division									
*Barishal*	Ref	0.29 (0.12–0.7)	0.006	0.37 (0.04–3.6)	0.395	0.24 (0.02–3.21)	0.278	1.33 (0.01–188.63)	0.911
*Chattogram*	Ref	1.10 (1.02–1.18)	0.014	1.7 (1.6–1.81)	0.000	1.36 (1.2–1.54)	0.000	1.54 (1.3–1.82)	0.000
*Dhaka*	Ref	0.64 (0.56–0.72)	0.000	0.72 (0.66–0.77)	0.000	0.55 (0.46–0.66)	0.000	0.43 (0.36–0.52)	0.000
*Khulna*	Ref	0.77 (0.72–0.83)	0.000	0.86 (0.8–0.93)	0.000	0.85 (0.73–0.98)	0.023	0.91 (0.78–1.07)	0.256
*Mymensingh*	Ref	1.28 (1.06–1.55)	0.011	3.09 (2.69–3.56)	0.000	4.55 (3.99–5.18)	0.000	7.49 (6.31–8.88)	0.000
*Rajshahi*	Ref	0.00 (0.00–0.00)	0.000	0.03 (0.01–0.06)	0.000	0.14 (0.07–0.28)	0.000	0.05 (0.01–0.23)	0.000
*Rangpur*	Ref	0.22 (0.15–0.33)	0.000	1.21 (0.97–1.51)	0.096	1.37 (0.75–2.5)	0.308	1.73 (0.91–3.27)	0.093
*Sylhet*	Ref	0.79 (0.72–0.88)	0.000	0.75 (0.71–0.79)	0.000	0.70 (0.62–0.79)	0.000	0.64 (0.56–0.72)	0.000

CI – confidence interval, DH – District Hospital, IRR – incidence rate ratio, MCH – Medical College Hospital, UHC – Upazila Health Complex

**Figure 4:**
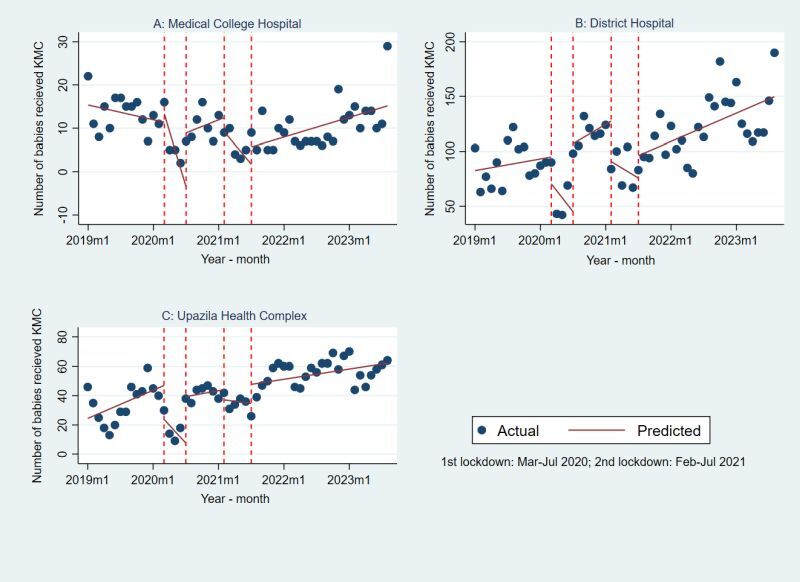
Segmented regression using interrupted time series of number of Kangaroo Mother Care (KMC) by type of facility. **Panel A.** Medical College Hospital. **Panel B.** District Hospital. **Panel C.** Upazila Health Complex.

**Figure 4** presents the fitted segmented regression using Poisson link for monthly trend of KMC across different phases of COVID-19 based of lockdown periods by three types of facility. During first lockdown the number of KMC reduced significantly in all types of facilities (**Figure 4**, panels A–C). The IRR was 0.69 (95% CI = 0.59–0.8), 0.89 (95% CI = 0.82–0.96) and 0.61 (95% CI = 0.49–0.77) for Medical College Hospital, District Hospital and Upazila Health Complex respectively. Between lockdown the change of KMC utilisation was not significant at Medical College Hospital but the number increased at district (IRR = 1.19; 95% CI = 1.11–1.27) and decreased at Upazila level health facilities (IRR = 0.77; 95% CI = 0.67–0.87) compared to pre-COVID-19 period. After COVID-19 period the District Hospitals’ KMC utilisation increased 1.21 times with 95% = 1.03–1.42. **Table 2** presents the details of the IRR and 95% CI associated with **Figure 4**.

**Figure 5:**
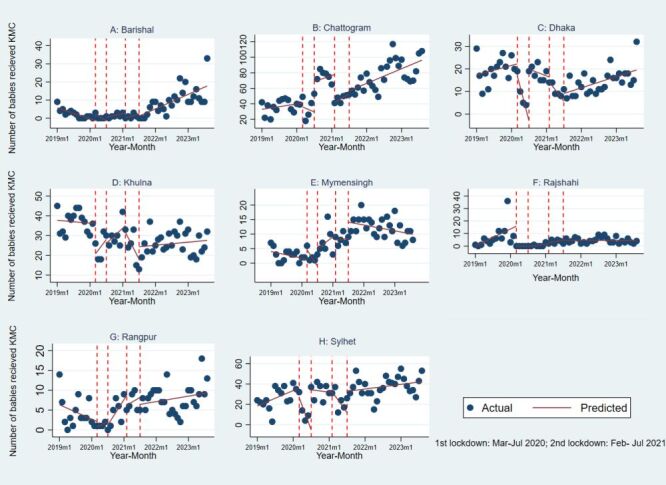
Segmented regression using interrupted time series of number of Kangaroo Mother Care (KMC) by eight divisions of Bangladesh. **Panel A.** Barishal. **Panel B.** Chattogram. **Panel C.** Dhaka. **Panel D.** Khulna. **Panel E.** Mymensingh. **Panel F.** Rajshahi. **Panel G.** Rangpur. **Panel H.** Sylhet.

**Figure 5** presents the fitted segmented regression using Poisson link for monthly trend of KMC across different phases of COVID-19 based of lockdown periods by eight divisions in Bangladesh. During first lockdown, all divisions had reduced KMC utilisation except Chattogram (IRR = 1.1; 95% CI = 1.02–1.18) and Mymensingh (IRR = 1.28; 95% CI = 1.06–1.55). In Mymensingh the IRR increased significantly at all phases of COVID-19 compared to pre-COVID-19. During the post-COVID-19 period the IRR was 7.49 with 95% CI = 6.31–8.88. At Dhaka the KMC utilisation reduced significantly during post-COVID-19 period with IRR = 0.43; 95% CI = 0.36–0.52. **Table 2** presents the details of the IRR and 95% CI associated with **Figure 5**, panels A–H. 

## DISCUSSION

Our analyses reveal the impact of COVID-19 on KMC service utilisation in public health facilities in Bangladesh through an interrupted time series analysis, shedding light on the pandemic's shock, health care system responses, and resilience.

In the pre-COVID-19 period (14 months), the study facilities provided an average of 137 KMC instances per month. During the subsequent first lockdown scenario (five months), KMC provision dwindled to an average of 97 instances per month, a clear reflection of the pandemic's disruptive influence. This study identified an upward trend in KMC utilisation during the pre-COVID-19 period, but with the onset of the COVID-19 pandemic and subsequent lockdown measures, KMC coverage experienced a significant decline. Specifically, our research indicates a 20% reduction in KMC utilisation during the first lockdown compared to the pre-COVID-19 timeframe. These findings are consistent with prior studies that have documented the initial effects of the COVID-19 pandemic on essential health care services [32–39]. A systematic review yielded a similar pattern, indicating a decrease of approximately one-third in health care utilisation during the pandemic [40]. A scoping review on one hundred and seventy studies also reported that the COVID-19 pandemic negatively affected the inpatient and outpatient health services utilisation in worldwide [39]. The scoping review highlighted potential explanations, as mentioned by the authors, which encompassed reduced health facility operating hours due to national lockdowns and curfews, limited availability of human resources within health care facilities, and reluctance among individuals to seek care owing to concerns about contracting the virus [39]. During the lockdown, certain pregnant women who wished to have facility-based births encountered obstacles primarily related to transportation restrictions [41,42]. Additionally, others experienced difficulties in accessing health care facilities [32]. As a result of these challenges, the rates of facility-based births decreased, and there was a noticeable decline in the utilisation of KMC. A study conducted in Bangladesh highlighted that in many facilities, outdoor health care services were effectively halted, and hospitals actively discouraged patient visits unless they were deemed absolutely essential, as a preventive measure to curb the spread of the virus [43]. Furthermore, a subgroup of women who had given birth at a health care facility may have opted for early discharge as a precautionary measure to reduce the risk of COVID-19 infection [44]. This choice led to a decrease in hospital stays and a corresponding decline in the utilisation of KMC.

However, as the health care facilities navigated the challenging landscape of COVID-19 and its associated lockdowns, during the 'Between Lockdown' scenario (six months), a spontaneous increase in KMC provision to an average of 172 instances per month was noted. Notably, KMC utilisation during the second lockdown (six months), while still lower than the pre-pandemic levels, exceeded the initial lockdown period, indicating a learned response from previous experiences. During the 'Between Lockdown' scenario KMC utilisation increases significantly by 5% and during the second lockdown only 1% reduction was observed compare to the pre-COVID-19 timeframe. This shift can be attributed to reduced apprehension among pregnant women and mothers regarding COVID-19 [43], as well as the fact that all health care services were fully operational [43], and transportation restrictions were relatively less stringent than during the initial lockdown. Possible reason for the positive response by the health care facility might be due to the measures taken by the Government of Bangladesh (GoB) like diagnosis of suspected cases, quarantine of doubted people and isolation of infected patients, local or regional lockdown, increasing public awareness, financial packages for doctors, nurses and health workers and social distancing to combat the COVID- 19 [45]. Additionally, the GoB implemented various initiatives to boost KMC utilisation, which encompassed online training programmes for health care professionals and rigorous monitoring measures.

As the study transitioned into the post-COVID-19 period (25 months), KMC utilisation rebounded, with the facilities providing an average of 185 KMC instances per month. This recovery showcases the health care system's remarkable resilience in adapting to and ultimately overcoming the disruption posed by the COVID-19 pandemic. In the post-COVID-19 period, there was a notable 8% increase in KMC utilisation compared to the pre-COVID-19 timeframe. This can be attributed to reduced fear among pregnant women and mothers concerning COVID-19, resulting in a return to more normalised health care-seeking behaviour [43]. The absence of transportation restrictions and the prompt treatment of emergency patients played a crucial role [43]. Furthermore, the Government of Bangladesh (GoB) continued to facilitate online training for health care professionals, further contributing to the rise in KMC utilisation.

The impact of the COVID-19 pandemic on KMC utilisation was widespread, affecting various facility types and divisions. When considering different facility types, the situation at DH mirrored the overall trend, but MCH and UHC facilities showed a different pattern. Following the COVID-19 period, the average monthly KMC utilisation in MCH facilities was lower than the pre-COVID-19 period. It is important to note that this discrepancy may be attributed to the limited number of MCH facilities included in this study. In terms of divisions, Dhaka, Khulna, and Rajshahi exhibited a contrary trend to the overall results, with the average monthly KMC utilisation post-COVID-19 being lower than the pre-COVID-19 period. Again, this divergence may be influenced by the small number of facilities from these divisions included in the study. Therefore, further research is recommended to delve deeper into the variations among facility types and divisions.

### Strengths and limitations

Our study stands as the initial exploration in Bangladesh to assess the impact of COVID-19 on KMC utilisation. Utilising 58 months of data, we were able to observe the shock, response, and subsequent resilience. However, our study does hold certain limitations. To maintain a consistent and adequate data set, a minimum criterion was set for facility inclusion, resulting in the analysis encompassing only 46 health facilities across Bangladesh. Consequently, cautious interpretation is warranted, especially concerning division and facility-specific results. Moreover, private health facility and NGO hospitals or clinics rarely report to DHIS2, hence it lacks generalisability. Additionally, the absence of diverse independent variables and qualitative data restricts a comprehensive understanding of recipient characteristics, KMC quality, subjective experiences, and health care providers' responses. Lastly, due to the study's design limitations, the study was not able to report the underlying reasons for the response of health care systems.

## CONCLUSIONS

Our analysis demonstrates the impact of COVID-19 on KMC utilisation. The significant drop in KMC instances during the initial lockdown period indicates the immediate impact of the pandemic on essential maternal and child health care services. Nevertheless, the subsequent phases demonstrated the health care system's adaptive response, showcasing learning and adaptation, notably evidenced by increased KMC uptake observed during the periods between lockdowns. In conclusion, Bangladesh exhibited resilience in combating the challenges posed by COVID-19, subsequently rebounding and revitalising KMC utilisation back on track. Further research endeavours are imperative to delve into the underlying causes derived from lessons learned during lockdown periods. Understanding these insights will fortify strategies aimed at effectively combating potential future pandemics.
